# Oral Cancer Screening by Artificial Intelligence-Oriented Interpretation of Optical Coherence Tomography Images

**DOI:** 10.1155/2022/1614838

**Published:** 2022-04-23

**Authors:** Kousar Ramezani, Maryam Tofangchiha

**Affiliations:** Department of Oral and Maxillofacial Radiology, Dental Caries Prevention Research Center, Qazvin University of Medical Sciences, Qazvin, Iran

## Abstract

Early diagnosis of oral cancer is critical to improve the survival rate of patients. The current strategies for screening of patients for oral premalignant and malignant lesions unfortunately miss a significant number of involved patients. Optical coherence tomography (OCT) is an optical imaging modality that has been widely investigated in the field of oncology for identification of cancerous entities. Since the interpretation of OCT images requires professional training and OCT images contain information that cannot be inferred visually, artificial intelligence (AI) with trained algorithms has the ability to quantify visually undetectable variations, thus overcoming the barriers that have postponed the involvement of OCT in the process of screening of oral neoplastic lesions. This literature review aimed to highlight the features of precancerous and cancerous oral lesions on OCT images and specify how AI can assist in screening and diagnosis of such pathologies.

## 1. Introduction

Medical imaging is an inseparable part of medical diagnosis and plays a pivotal role in cancer screening and follow-up of treatments. In the specific field of oncology, imaging provides valuable anatomical and functional information that can preciously improve the results of screening, diagnosis, treatment, and follow-up [1]. Computed tomography (CT), magnetic resonance imaging (MRI), ultrasonography, positron emission tomography, single photon emission CT, and other modalities are utilized to detect tumoral changes. However, none of these modalities can address all aspects of a cancer diagnosis. CT, MRI, and ultrasonography provide structural information about the tumors, such as their location and extension; meanwhile positron emission tomography and single photon emission CT reveal functional and molecular information [2]. Besides, there are still demands to improve spatial and contrast resolutions of these modalities to provide more accurate information [1, 3].

Early diagnosis of tumoral changes not only ensures appropriate timing for surgical intervention and subsequent treatment and increases the survival rate but also decreases postsurgical morbidity, especially in invasive and malignant tumors, because less tissues are involved. Definite cancer diagnosis relies on histopathological assessment that requires tissue preparation and is time-consuming. Moreover, in large tumors, several samples from different sites need to be excised. Besides, in the process of surgical removal of a tumor, surgeons need to examine the excised margins several times to ensure leaving clear and tumor-free margins. In such cases, immediate and precise histopathological examination may not be practically possible [4]. Artificial intelligence (AI) could be important as an auxiliary diagnostic tool due to the fact that intraoperative frozen sections are not ideal outcome predictors in certain locations [5]. Therefore, the use of computer science may be of assistance to noninvasively improve the accuracy of diagnosis.

Approximately 300,000 new cases of oral cancer are diagnosed annually worldwide which are responsible for over 145,000 deaths per year. Oral cancer ranks the sixth most common cancer worldwide, with an increasing incidence rate but constant survival rate during the past decades because of delayed detection and reliance on traditional screening methods. The importance of early detection of oral cancer is further understood when comparing the 5-year survival rate of 80–90% in case of early diagnosis and treatment with 30% rate in cases diagnosed in advanced stages [6, 7]. Oral leukoplakia, erythroleukoplakia, and verrucous hyperplasia are precancerous lesions with the potential to transform into squamous cell carcinoma (SCC) [8]. SCC accounts for 90% of all oral malignancies [9]. Biopsy is the gold standard for oral cancer diagnosis; however, it is painful, and, in case of extensive or multiple lesions, site and size selection for surgical removal of biopsy sample is critical and sometimes confusing [10]. Moreover, the prepared histological specimen may not reflect the identity of the whole lesion due to lesion heterogeneity. Adjunctive methods to aid the clinicians in selecting the best site for biopsy decrease false-negative results [11].

Optical coherence tomography (OCT) is a noninvasive real-time imaging modality that delivers three-dimensional (3D) high-resolution microscale images (axial and lateral resolutions of 13–17 *μ*m and 17–22 *μ*m, respectively). An 8 *μ*m axial resolution and a 2 *μ*m axial and lateral resolution are reported as state of the art. OCT is fast, repeatable, and well tolerated by patients [12–14]. It has several potential applications in oncology. Real-time nondestructive high-resolution radiation-free OCT images make it an ideal modality for screening of neoplastic tissue changes. Moreover, it can aid in targeted biopsy, intraoperative surgical margin and lymph node histopathological assessments, and postoperative evaluation of treatment response, resulting in more successful tumor resection and improved survival rate [4]. Evidence shows that OCT images reveal helpful information for detection of early-stage oral cancer [15].

AI is affecting most aspects of human life; image-based medical diagnosis is not an exception. Screening of oral lesions relies on subjective interpretation of clinical features, which considerably varies in accuracy, sensitivity, and specificity as reported in the literature. Dentists are at the frontline of encountering cancerous lesions and have variable levels of diagnostic accuracy in detection of different lesions. Delayed referral to a specialist is a major cause of poor outcome of management of oral premalignant lesions [16–19]. AI enables easy access to specialized diagnosis specially in patients who cannot be referred to more equipped medical centers because of residing in remote localities or due to high transportation costs. Free software utilization can improve the accuracy of surgical treatment planning for oral cancer as well [20]. The recent COVID-19 pandemic proved the importance of technologies that eliminate the necessity of physical presence to receive services [21, 22]. The screening process for oral lesions currently lacks an accurate, nonsurgical, and reproducible imaging approach. Unfamiliar images for dental clinicians and a software environment that is difficult to interact for the operators are the main barriers against the widespread use of OCT for detection of oral lesions, despite its unique characteristics and high diagnostic value [16].

This literature review aims to summarize the features of oral precancerous and cancerous lesions on OCT images for medical and dental practitioners who are involved in diagnosis and treatment of these lesions and highlight how AI can improve the screening and diagnosis of such lesions.

## 2. Methods

A comprehensive search was conducted in PubMed for articles published in English up to August 28, 2021, using this query: (((optical coherence tomography) OR (OCT)) AND ((oral cancer^*∗*^))) AND ((artificial intelligence) OR (machine learning) OR (deep learning) OR (convolutional neural network)). The inclusion criteria included studies that investigated oral precancerous or cancerous lesions on OCT images by any AI algorithm. Relevant articles were initially included based on their title and abstract and subsequently by their full text. The reference lists of relevant articles were also explored to find possibly missed articles.

### 2.1. OCT

#### 2.1.1. Mechanism of Action

OCT uses a partially coherent near-infrared light beam of variable wavelength to image partially transparent tissues. The infrared wavelength (780–1550 nm) is a spectrum of light with deepest penetration into biological tissues (biological window) [23]. The beam reflected from the tissue layers produces an electric signal which can be detected afterwards. The term “tomography” implies the provided sections of the imaged object [24].

OCT consists of a broadband light source, an imaging system, a transducer, a data processor, and a computer to control the entire scanning process and image visualization [24]. The imaging principle of OCT is similar to that of ultrasonography. They both measure the backscattered beam emitted to the tissues, but, due to the differences in the wavelength and speed of light and acoustic waves, the penetration depth and the resolution they present are different. The mechanism of receiving the backscattered beam is fundamentally different as well [24]. The velocity of light is much higher than that of ultrasound waves; thus, measurement of time delay is impractical. Therefore, OCT utilizes an interferometer to calculate the pathway difference of light [25, 26]. The emitted beam is divided into the reference beam and sample beam which colligate again after reflection from the reference mirror and tissues, respectively. A photodetector or spectrometer records the interferences and digitizes them to be depicted graphically on a computer [27, 28]. The optical interfaces backscatter the emitted light with a time delay that is retrieved by Fourier transformation and used to calculate the distance between the optical reflections of tissue layers through interferometry resulting in A-scan sections. B-scan or longitudinal images employ a series of A-scans along a line on the *x*-axis and *z*-axis to create 2D views. C-scans or enface images are also 2D views obtained from the *x*-axis and *y*-axis. Volumetric data are reconstructed by 2D scanning of the layers [28–30].

The optical characteristics of a sample dictate the optical path and penetration depth of OCT beam, and OCT images reflect the coefficient of transmission information of a sample [31]. The penetration depth declines as the density of the material increases [32]. Translucency of the medium determines the penetration depth as well [2, 19]. The axial resolution is defined by the wavelength and bandwidth of the light source [33].

OCT devices can be time-domain or frequency-domain devices based on their reference arm optics. The frequency-domain devices are of two types as well, spectral OCT and swept source OCT, based on the receiving compartments and output properties. The swept source OCT uses ultrahigh speed (kilohertz wavelength, center wavelength 1300 nm) laser beam which enhances the sensitivity of the system, penetration depth, resolution of the system, and scanning rate (1 second or less imaging speed), resulting in a shorter acquisition time [31, 34–37]. In the swept source OCT, the axial and transverse resolutions are determined by linewidth of the laser beam and focus spot size, respectively [23].

OCT can accompany probes and catheters to image internal organs and structures [4]. It should be noticed that mechanical compression of OCT probe on the soft tissue alters the optical properties of the tissue layers, increases the contrast between the layers, and decreases the thickness of the layers [38].

Although OCT is fundamentally a label-free modality, different contrast agents are studied to target specific cells or tissues including magnetic nanoparticles, gold nanoparticles, and encapsulating protein-shell microspheres [39–44]. Vessels could be detected on OCT images based on signal changes of light without additional contrast agents or dyes [45]. A study conducted to image the sublingual microcirculation found OCT to be suitable for this purpose [46]. A significant correlation has also been reported between histological slides and OCT images [10].

#### 2.1.2. Dental Applications

OCT has proven its novel capabilities in some fields of medicine such as ophthalmology and cardiology; however, it has not been widely used in dentistry. OCT has been employed as a valuable tool for assessment of the anterior part of the visual pathway, optic nerve characterization, and visualization of cellular layers of macula [47]. Functional blood vessels of the eyes can be three-dimensionally reconstructed by OCT angiography based on its ability to detect moving red blood cells, which induce variations in the OCT signal [48]. OCT has been widely studied for detection of skin cancers and cutaneous inflammatory diseases based on its ability in imaging of skin tissue layers and substructures [49–51]. The hard and soft tissues of the maxillofacial region need a wide range of OCT rays. The scattering properties of wavelengths below 1000 nm match the dimensions of tissue particles, resulting in more efficient imaging. The tissues with higher water content dissipate the energy of the beam more; consequently, hard and soft tissues require adjusted wavelengths to obtain the best images [52]. Nontransparent tissues limit the penetration of OCT beam because of absorption and scattering effects [4]. OCT has higher penetration depth in comparison with the majority of optical imaging modalities [53–55].

Different sites of the oral cavity and hard-to-reach areas require customized applicable probes. For some pathological lesions, greater depth and a larger field of view may be required for a more comprehensive assessment of the tissues [56, 57].

The first designed OCT device for dental applications was hoped to be used for imaging of gingival margins, periodontal attachments, and pockets [58]. OCT has been used for evaluation of caries, propagation of demineralization or remineralization process, cracks, wear, erosion, deformations, age-related changes, restoration defects, root canal system, detection of pulp horns and isthmuses, sealing efficacy of cements, and evaluation of penetration depth of different materials into the tooth structure [59–65]. A unique superiority of OCT over the conventional X-ray examination for caries detection is visualization of incipient caries that could not be detected radiographically without radiation exposure. Radiography cannot distinguish active caries from arrested caries [24, 66]. Moreover, enamel and dentin could be easily differentiated on OCT images due to their different optical properties [12]. OCT shows promising results in pediatric dentistry for incipient caries detection due to its real-time and noninvasive nature [67]. In maxillofacial surgery, OCT can be used for soft tissue assessment and differentiation of normal tissue from dysplastic and malignant changes. Some studies used OCT to evaluate the periodontal tissues, peri-implant tissues, radiation-induced oral mucositis, and bullous lesions [68–72].

#### 2.1.3. Oncological Applications

OCT does not have a large field of view or high penetration depth, but its microresolution and high soft tissue contrast due to differences in scattering properties make it an ideal nonsurgical modality to spatially differentiate cell layers and tissues. It has been widely used in oncological studies in vivo and in vitro [4, 73–75]. Visualization of microanatomy is not the only domain that OCT can shine in; it has been used to assess several cellular dynamics and cell processes that occur in premalignant and malignant tissues [76, 77]. Several OCT devices and probes have been commercialized for oncological applications [4].

Three-dimensional cell colonies are developed to aid in investigation of tumorigenesis mechanism and drug response with no need for animal models. OCT can monitor such samples periodically and repeatedly. It has been demonstrated that OCT has the ability to detect dead cells based on their scattering properties [78]. OCT is a good modality to monitor neoplastic changes in cellular scale and treatment response in 3D culture studies [76].

#### 2.1.4. Cancer Indicators

Measurement of epithelial thickness on OCT images is valid, reliable, and practicable. The normal epithelial thickness is 75–550 *μ*m in different sites and can be imaged by OCT with 2–3 mm penetration depth and 10–12 *μ*m resolution [38, 79, 80]. The oral mucosa on OCT images is described as a hyporeflective epithelium underlined by the basement membrane and a hyperreflective lamina propria beneath them, containing blood vessels and minor salivary glands [10]. The normal oral epithelium has a homogenous distribution of cells that are uniform in size and nucleus/plasma ratio, while this arrangement is impaired by cancer clusters and nests with variable cell sizes and nucleus/plasma ratio in cancerous epithelium [8].

Neoplastic changes are characterized by cells that are abnormal in shape and size and have enlarged nuclei. Such changes at the cellular and subcellular levels change the optical scattering properties of OCT, which enhances their detection. Some important histological indicators of malignancy in the epithelial tissue include expanded dysplastic cells, irregular epithelial stratification accompanied by broadened rete pegs, basal hyperplasia, and elongated papilla core [10, 81]. Dysplastic cells in the epithelium produce a dispersed speckle pattern on OCT B-scans [33]. Speckle formation is inevitable on OCT images because of the heterogeneous nature of the biological tissues which interferes with the optical beam in various levels [82].

Researchers have tried to find some indicators to differentiate intact, premalignant, and neoplastic tissues in epithelial mucosa, subepithelial tissue, and basement membrane of oral mucosa on OCT images. Thickening of basement membrane is a sign of tumor invasion and can be considered as an indicator of malignant changes. In a previous study, the mean epithelial layer thickness was the highest in microinvasive carcinoma, followed by carcinoma in situ, dysplasia, and benign lesions [83]. Another study confirmed increased thickness of epithelial layer after dysplastic changes, albeit the boundary between the epithelium and lamina propria, unlike SCC, could be delineated [84]. Tsai et al. considered epithelial thickness, the standard deviation of A-mode scan intensity profile, and the exponential decay constant of spatial-frequency spectrum of the A-mode scan profile as indicators to distinguish benign and malignant oral lesions [8, 84, 85]. They found that, in abnormal oral mucosal lesions, the standard deviation increased, the decay constant of the spatial-frequency spectrum decreased, and the epithelium thickness increased [8]. Neoangiogenesis, surface integrity, surface profile (even or uneven), epithelial homogeneity, loss of stratification in squamous epithelium, and tissue vascularization are other indicators as well [10, 86]. Neoplastic transformation can cause stromal changes, alter collagen and other extracellular components, or induce fibroblast proliferation [87, 88].

A clear boundary between the epithelium and lamina propria could not be identified on B-mode scans of cancerous lesions [8]. The epithelial thickness and basement membrane integrity are valid indicators to differentiate normal and dysplastic tissues, as well as invasive carcinoma [89]. The epithelial thickness increases prior to disappearance of lamina propria due to invasion of cancer cells [8].

The epithelial and subepithelial changes following dysplastic transformation result in stronger light scattering and fluctuation of spatial distribution. The mean intensity of spatial reflection is greater in dysplastic oral epithelium in comparison with normal tissue. Moreover, collagen deposition in lamina propria results in a reduction in SD level [85].

There is a distinctive contrast in signal intensity between the epithelium and lamina propria (bright epithelium and brighter lamina propria) in oral premalignant tissues; subsequently, this boundary can be precisely identified [85]. Lee et al. [85] used computer analysis to automatically differentiate normal and precancerous oral mucosa. They successfully plotted the boundary between the epithelium and lamina propria, measured the epithelial thickness, and estimated the range of dysplastic cell distribution.

#### 2.1.5. AI

There are some drawbacks related to the oncological applications of OCT such as limited penetration depth, limited area and volume of scans, demand for higher resolution to visualize more cellular and subcellular details, too much noise, difficult image interpretation, and the training required for image interpretation [31]. Interpretation of OCT images is extremely operator-dependent, because there is no defined comprehensive and precise standard for interpretation of OCT images [30]. Interpretation of OCT images requires training and expertise, and since the configuration of OCT images is basically different from the conventional images, even medical imaging experts have difficulty in reading the OCT images. AI and machine learning algorithms can assist in interpretation of OCT images, providing fair and equal accessibility to an automated professional diagnosis with high accuracy [90]. Cutting-edge technologies introduced to the biomedical field might have too much presentable information and data, but as long as these datasets could not be precisely and efficiently translated to a clinical insight in a timely manner to affect the diagnosis and treatment outcome, they would be a waste of cost and time [91]. Deep learning algorithms and AI have not still found their appropriate clinical position despite their marvelous ability to increase the accuracy of interpretation and eliminate the efforts, cost, and time spent to train the operators [50].

Automatic processing of the features of OCT images not only saves the time of analyzing abundant volume of data but only will digitize the data that could not be interpreted subjectively [33]. Machine learning and deep learning are two subfields of AI. Machine learning algorithms need structured and labeled datasets; meanwhile deep learning algorithms generate their own subsets of data by identification of differences within layers of neural networks. Unlike the machine learning algorithms, deep learning algorithms require an abundant amount of data to build their network and perform their best [16]. Machine learning is fed by a large amount of ground truth manually labeled by the clinicians. Generation of expert-defined annotations is time-consuming and costly and is deteriorated by intergrader variability [92]. Deep learning has a multilayered convolutional neural network to learn and distinguish image features [93]. Deep learning enhances predictive accuracy by weight adjustment of data through a process called back-propagation [94]. It strengthens or weakens the weight of each synapse based on the input “answer” to reach the highest agreement. Deep learning diagnosis is built based on the multiblinded experts' decisions. If the introduced data to the algorithm are large enough, intergrader variability will not have a significant impact on evolution of training. A trained model might present unknown information, because it might recognize features that could not be detected by the operators [21, 95].

Artificial neural networks are machine learning algorithms made of associated working units known as neurons. The neurons are arranged in layers and acquire a value based on the activation frequency. When the neuron layers extend to more than 2–3 layers, it is known as deep learning artificial neural network. Deep learning convolution neural network is a model with multiple neural layers that administrate template-matching of input data with ground truth. Convolution neural network can be trained by continuous introduction of structured data and can upgrade its operation ability [96]. Convolutional neural networks are the most popular deep learning programs for analyzing visual figures [21]. Deeper number of convolutional layers and bridge connections between layers in deep learning algorithms bring about higher performance [82]. Even a minor sensitivity to variations in tissue characteristics might be sufficient for deep neural network architecture to diagnose alterations in the relevant image properties, a specialty that even an expert rater cannot reach visually [82]. Various neural network architectures are introduced for semantic segmentation process [97]. Manual image segmentation needs time and expertise and has variable reproducibility, and, in contrast to automated algorithms trained for this application, high-quality images are required [98].

Deep learning algorithms have shown promising results in ophthalmology such as macular edema detection, retinal thickness measurement, and retinal layer segmentation [95, 99–105]. They have been successfully used for compartmentalization of retinal layers with statistics comparable to the human grader [106]. Automated detection of basal cell carcinoma on OCT images has shown promising results with excellent statistics of around 95% for accuracy, sensitivity, and specificity [90]. An automated segmentation algorithm can delineate the basement membrane and measure the epithelial thickness [107]. AI has been used to extract pathological features of CT, MRI, and endoscopy images [108–110]. Hwang et al. [21] designated a workflow for involvement of AI in the processing of OCT images for macula edema screening. Their workflow framework can be generalized to oral lesion screening on OCT images.

A marvelous preponderance of image translation by AI is that AI can quantify variations which could not be even detected by inspection. Specific characteristics of the tissue texture could not be quantified by the naked eye. Moreover, AI is able to integrate multiple data variables including imaging, geographical, clinical, pathological, and electronic health data as well as risk factors, resulting in a more comprehensive and analytical diagnosis [16].

Appending a diagnostic algorithm to the OCT system eliminates the operator training considerations as well as inter- and intraoperator diagnostic errors and nullifies subjectivity in interpretation of images. It can speed up the analysis of large amounts of datasets as well.

**Figure 1:**
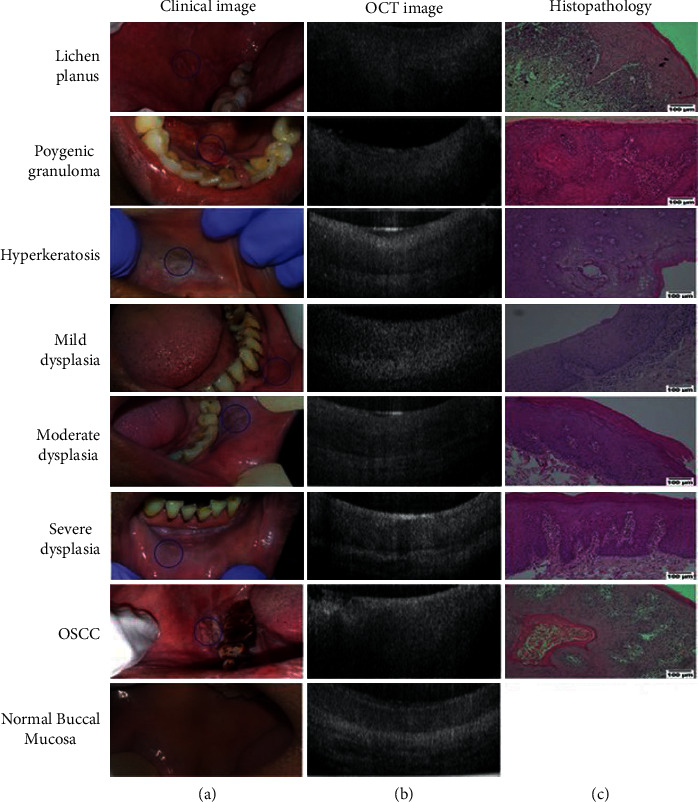
Clinical, OCT, and histological images. Clinical (a) and OCT (b) images were captured from all subjects, and biopsy samples were collected (wherever indicated) and assessed histopathologically (c). Histological images were taken at 100x resolution (scale bar = 100 *μ*m) using Nikon DSFi2 and NIS elements D4 20.0. The nondysplastic lesions shown were histologically diagnosed with lichen planus, pyogenic granuloma, and hyperkeratosis. Normal buccal mucosa images were taken from a healthy volunteer without any habit history. Representative images of all dysplastic grades and a buccal oral squamous cell carcinoma (OSCC) are also depicted (image courtesy of https://bit.ly/316d1S1).

There are few studies that utilized computational algorithms for normal or cancerous tissue characterization [8, 85]. Lee et al. [85] used standard deviation of the intensity of OCT images to identify normal and dysplastic oral mucosa. Pande et al. [33] described algorithms to characterize morphological features on OCT B-scans of hamster cheek pouch. They evaluated aberration of layered structure of the epithelium and epithelial thickness, which are indicators of malignant transformation. Their algorithm showed 78.5%, 76.6%, and 87.8% sensitivity for diagnosis of benign, precancerous, and cancerous lesions, respectively, and 87.6%, 86.2%, and 94.3% specificity for the aforementioned lesions, respectively. These results were strongly suggestive of automated oral cancer detection on OCT images. James et al. [111] implemented artificial neural networks and a support vector machine model to annotate image features of OCT images obtained from the normal oral mucosa and benign and malignant lesions (Figure 1).

The statistics demonstrated that OCT-based diagnosis of malignant and dysplastic oral lesions integrated with AI had comparable results (93–96% sensitivity and 74–49% specificity) to the biopsy. Heidari et al. [112] deployed a convolution neural network to discriminate normal and abnormal head and neck mucosa on 3D OCT images. They reported 100%, 70%, and 82% sensitivity, specificity, and accuracy, respectively, for identification of cancer-positive images.

A cloud-based platform has been launched for remote machine learning of OCT image analysis that could be employed for analysis of tumor images. Open access to this platform will nourish the algorithms with more diverse data, leading to enhanced performance [106].

### 2.2. Limitations

High cost and limited availability limit the extensive application of OCT in the clinical setting, leading to less available necessary data to enrich and enhance AI algorithms. Affordable OCT devices not only provide accessibility to this modality but also would supply the image analysis algorithms with more datasets. Since the diagnosis of oral cancer and precancerous lesions, interpretation of OCT images, and development of AI algorithms for automated OCT image interpretation require profound expertise, qualified teams with experts from all related fields are required to collaborate interactively to achieve the desirable results. These requirements decrease the pace of employing AI algorithms in oral cancer screening and diagnosis by OCT images. Further studies are required to analyze and improve the efficiency and accuracy of AI algorithms for detection of cancerous changes before we could use such automated interpretation systems in the clinical setting.

## 3. Conclusion

AI algorithms have rendered hopeful outcomes in interpretation of OCT images of oral mucosa and discrimination of normal oral epithelium from precancerous and cancerous lesions. Progressive evolution of AI algorithms for interpretation of OCT images (which requires continuous data feed as ground information) paves the way towards automated oral cancer screening by OCT, even though it might be a long road to bring the integration of OCT and AI into the clinical setting. In addition to the need for further studies to provide OCT imaging data for AI algorithms, the existing challenges such as standardization of labeling, validation of automated interpretations, and development of infrastructures for application of AI in oral cancer screening and diagnosis must be addressed to enable the application of AI in OCT for the aforementioned purposes and enhance early detection of oral mucosal cancerous changes with no need for physical presence of experts.

## Data Availability

This review article is supported by the data of previously reported in the studies and data sets which were cited.
